# Naloxone reversal of fentanyl-induced respiratory depression versus naloxone-induced precipitated withdrawal: an *in silico* utility model

**DOI:** 10.3389/fphar.2026.1944044

**Published:** 2026-09-10

**Authors:** Albert Dahan, Jack D. C. Dahan, Erik Olofsen, Maarten Van Lemmen, Monique Van Velzen, Elise Sarton, George Dungan, Thomas L. Miller, Robert B. Raffa, Chris Martini, Marieke Niesters

**Affiliations:** 1 MediD Consultancy Group, Amsterdam, Netherlands; 2 Centre for Human Drug Research, Leiden, Netherlands; 3 Enalare Therapeutics Inc., Mullica Hill, NJ, United States; 4 Amsterdam University Medical Center, Amsterdam, Netherlands; 5 Leiden University, Leiden, Netherlands; 6 Erasmus Medical Center, Rotterdam, Netherlands; 7 University Medical Center Utrecht, Utrecht, Netherlands

**Keywords:** fentanyl, model, opioid-induced respiratory depression (OIRD), utility (theory), withdrawal

## Abstract

**Background:**

Naloxone reverses opioid-induced respiratory depression (RD) but may precipitate withdrawal in opioid-dependent individuals. Although both have been studied separately, their concentration-response relationships have not been described within a single model. We developed a mechanistic pharmacokinetic/pharmacodynamic simulation model combining an established model of naloxone-induced reversal of fentanyl-induced RD with a novel mechanistic model of precipitated withdrawal based on normalized μ-opioid receptor activation.

**Methods:**

Utility was defined as the probability (P) of respiratory reversal minus the weighted probability of precipitated withdrawal, P(reversal) − k·P(withdrawal), where k denotes the relative weight assigned to withdrawal. A withdrawal threshold parameter *w* was introduced to reflect the fractional decrease in μ-opioid receptor activation required to precipitate withdrawal. *w* was calibrated using published naloxone-withdrawal data. Simulations allowed evaluations across varying naloxone concentrations, overdose severities, withdrawal thresholds and weighting factors.

**Results:**

The model reproduced the expected concentration-response relationships for respiratory depression, withdrawal, and their combined utility. Utility increased with higher withdrawal thresholds and decreased with greater withdrawal weight. Calibration against clinical data yielded a preliminary *w* estimate = 0.30 (95%CI 0.16–0.40). At this value of *w* at k = 1 (a neutral model reference of equal weight to reversal and withdrawal), the utility was negative over the naloxone concentration range of 10–30 ng/mL, indicating that modeled probability of precipitated withdrawal exceeded that of model-defined respiratory reversal over this range. At k < 1, the utility became positive.

**Conclusion:**

The proposed model quantitatively links two pharmacodynamic consequences of competitive μ-opioid receptor antagonism and generates testable hypotheses regarding the receptor activation threshold underlying precipitated withdrawal. The presented utility function is an experimental modeling exercise. In life-threatening opioid overdoses, restoration of ventilation, prevention of hypoxic injury and survival take priority over avoidance of withdrawal (*i.e.,* set k < 1).

## Introduction

Opioid overdose remains a major public health challenge, with synthetic opioids such as fentanyl responsible for a rapidly increasing proportion of fatal overdoses worldwide (Volkow and Blanco, 2021). Death following opioid overdose is primarily caused by opioid-induced respiratory depression (OIRD), which may progress to apnea, hypoxia, cardiac arrest, and death if untreated (Strauss et al., 2024). Prompt administration of the competitive μ-opioid receptor antagonist naloxone reverses respiratory depression in most cases (Strauss et al., 2024). Naloxone is a World Health Organization essential medicine and has become the standard treatment for suspected opioid overdose in both community and healthcare settings (World Health Organization, 1983).

Despite its life-saving efficacy, naloxone administration is frequently accompanied by precipitated opioid withdrawal in opioid-dependent individuals (Buajordet et al., 2004; Monroe and Radke, 2023). Symptoms range from nausea, vomiting, diarrhea, sweating, cramps, confusion, restlessness, tremor, severe anxiety to agitation and aggression (Bluthenthal et al., 2020). Although precipitated withdrawal is highly distressing and may increase health risk, it is rarely life-threatening. It may nevertheless discourage people who use opioids from accepting naloxone treatment or remaining under medical supervision after overdose reversal (Gaddis and Watson, 1992; Neale and Strang, 2015; Bluthenthal et al., 2020). Still, fear of withdrawal should never delay life-saving respiratory support or administration of an available opioid antagonist in a life-threatening overdose (Gold et al., 2025).

Current naloxone dosing recommendations largely reflect empirical experience rather than a quantitative understanding of this benefit-harm balance (Utrilla et al., 2025). Previous studies have successfully characterized fentanyl-induced respiratory depression and its reversal by naloxone (Boom et al., 2013; van Lemmen et al., 2025a; van Lemmen et al., 2025b; van Lemmen et al., 2026). Likewise, several clinical and experimental studies have described the occurrence of precipitated withdrawal after naloxone administration (Gaddis and Watson, 1992; Buajordet et al., 2004; Neale and Strang, 2015; Monroe and Radke, 2023). To our knowledge, no single model currently combines respiratory rescue and precipitated withdrawal and allows their relative probabilities to be evaluated simultaneously across different overdose severities and naloxone exposures.

Utility analysis provides a concept for addressing contrasting outcomes. Originating from decision theory, utility functions quantify the balance between beneficial and adverse effects and have previously been applied in pharmacology to optimize analgesia while minimizing respiratory depression or other adverse drug effects (Sheiner and Melmon, 1978; Cullberg et al., 2005; Kharasch and Rosow, 2013; Habibi et al., 2017). Here, we used the utility concept as a modeling tool to compare the probability of model-defined respiratory reversal with the probability of precipitated withdrawal.

The mechanistic pharmacokinetic/pharmacodynamic (PK/PD) simulation model that we developed combines an established ventilatory model of fentanyl-induced respiratory depression with a novel mechanistic model of precipitated withdrawal based on normalized μ-opioid receptor activation. Rather than modeling the concentration-effect relationship, the model derives withdrawal from naloxone-induced competitive receptor antagonism. It links respiratory reversal and withdrawal to the same underlying pharmacodynamic process but allows acute opioid effect and withdrawal susceptibility to be represented by distinct parameters. We integrated these two components into a single utility function, which allows evaluation of the balance between respiratory reversal and withdrawal across a range of naloxone concentrations, overdose severities, and patient characteristics. Finally, we explored the influence of main model parameters and obtained a preliminary estimate of the withdrawal threshold from published clinical data.

## Methods

### Pharmacokinetic and pharmacodynamic models

**FIGURE 1:**
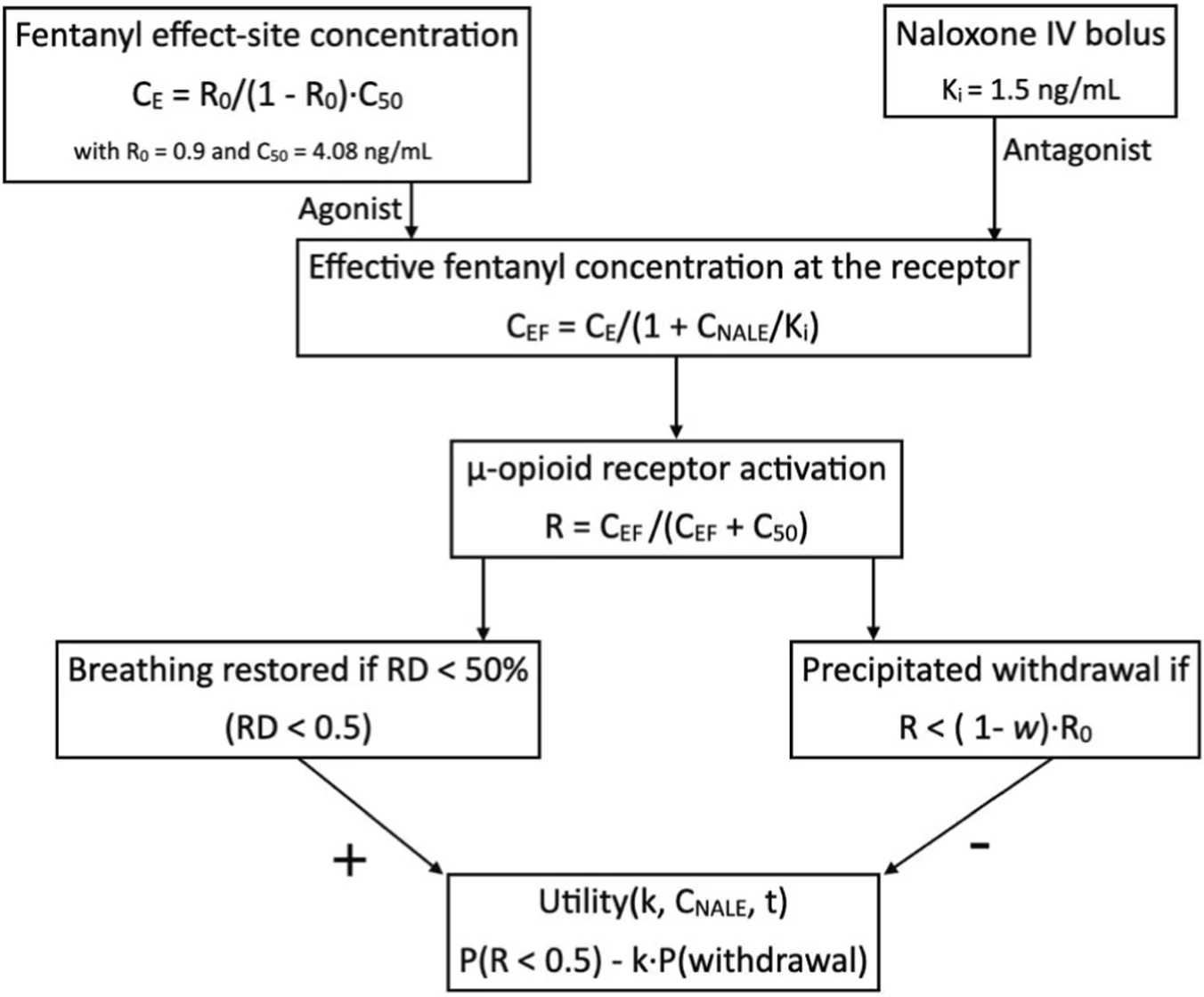
Schematic representation of the utility model of reversal of fentanyl-induced respiratory depression versus precipitated withdrawal. See the text and Equations 1–8 for explanation.

We performed an *in silico* population pharmacokinetic–pharmacodynamic (PK/PD) simulation study to construct a utility function describing naloxone reversal of opioid-induced respiratory depression (OIRD) in opioid-tolerant individuals, in which restoration of breathing represents the desired effect and precipitated withdrawal the undesired effect. No new human data were collected. The ventilatory PK/PD model has been described previously (Boom et al., 2013); a principal new component of the present study is a mechanistic withdrawal model based on normalized μ-opioid receptor activation. Figure 1 gives a schematic overview of the complete model.

Fentanyl disposition was described by a three-compartment model (Boom et al., 2013) with an effect-compartment linked by the hysteresis rate constant k_e0F_. Naloxone was modeled as a competitive µ-opioid receptor antagonist that reduces the effective opioid concentration at the receptor according to (Mu et al., 2024):

$$C_{\text{EF}}=C_{E}/\left(1+C_{\text{NALE}}/K_{i}\right)$$ (1)

where C_E_ is the fentanyl effect-site concentration, C_NALE_ is the naloxone effect-site concentration and K_i_ the naloxone inhibition constant. Naloxone does not alter the actual fentanyl concentration C_E_ but reduces the effective fentanyl concentration C_EF_ at the receptor that drives the pharmacodynamic response. Consequently, increasing naloxone concentrations reduce µ-opioid receptor activation, thereby reversing respiratory depression while simultaneously increasing the risk of precipitated withdrawal. Naloxone effect-site concentration was described using the hysteresis rate constant k_e0N_. See Table 1 for parameter values.

**TABLE 1:** Parameter values used in the simulations.

*Parameter*	*Value*	*Explanations*	References
Fentanyl pharmacokinetic and pharmacodynamic model parameters			
E_MAX_ (L/min)	19.9	Isohypercapnic ventilation at baseline	Boom et al. (2013)
C_50_ OUD (ng/mL)	4.1	Potency parameter in OUD	Algera et al. (2020)
γ	1	Steepness parameter	Boom et al. (2013)
ω^2^ (C_50_)	0.31	Inter-individual variability in C_50_ in the log-domain	Boom et al. (2013)
Naloxone pharmacokinetic and antagonism model parameters			
K_i_ (ng/mL)	1.5	Inhibition constant	Moss et al. (2020); Stolbach et al. (2023)
k_e0_ (min^-1^)	0.087	Blood-effect site rate constant	Van Lemmen et al., 2025b
Vd (L)	128	Apparent volume of distribution	FDA (2022)
k_e_ (min^-1^)	0.0077	Elimination rate constant	FDA (2022)
Range of withdrawal and utility model parameters inputted in the model			
*w*	0.3–0.7	Fraction drop in receptor activation causing withdrawal	This study: Calibrated to 0.3 (95% CI 0.16–0.40)
w*	0.3–0.5	Crossover value with withdrawal occurring at w < w*	This study: At R_0_ 0.9 w* = 0.44
k	0.25–4	Withdrawal probability weight in the utility function	This study
R_0_	0–0.97	Baseline normalized μ-opioid receptor activation	This study

Ventilation under isohypercapnic (CO_2_ clamped) conditions with an inhibitory sigmoid E_MAX_ model of the form (Boom et al., 2013)

$$\text{Effect}\left(t\right)=E_{\mathrm{MAX}}\;\cdot \;\left[1/\left(1+A\right)\right]$$ (2)

with

$$A={\left[C_{\text{EF}}\left(t\right)/C_{50}\right]}^{\gamma }$$ (3)

Baseline ventilation (E_MAX_) was fixed at 19.9 L/min, γ at 1 and C_50_ (the fentanyl C_E_ causing 50% respiratory depression) at 4.08 ng/mL, a proposed C_50_ for individuals with an opioid use disorder (OUD) and based on Algera et al. (2020). Inter-individual variability in C_50_ was log-normally distributed with variance ω^2^ = 0.31 (Table 1).

The respiratory outcome was model-defined reversal of respiratory depression, *i.e.,* residual respiratory depression below 50% of baseline. Under the sigmoid E_MAX_ model this is equivalent to C_EF_ < C_50_. This ventilation threshold is a model endpoint derived from Mann et al. (2022) and not a clinical definition of successful overdose reversal.

Withdrawal was modelled by assuming that opioid-tolerant individuals are physiologically adapted to µ-opioid receptor activation. Baseline normalized μ-opioid receptor activation (R_0_) represents the acute opioid effect before naloxone dosing and defined overdose severity. R_0_ it is not a measure of dependence. For a given R_0_, the corresponding fentanyl effect-site concentration follows from:

$$R_{0}=C_{E}/\left(C_{E}+C_{50}\right)$$ (4)

Following naloxone administration, normalized μ-opioid receptor activation becomes:

$$R=C_{\text{EF}}/\left(C_{\text{EF}}+C_{50}\right)$$ (5)

Precipitated withdrawal was assumed to occur when receptor activation decreased by more than a fraction *w* from its pre-naloxone baseline:

$$R\;<\;\left(1-w\right)\cdot R_{0}$$ (6)

where *w* is the single free parameter of the withdrawal model. Larger values of *w* indicate that a greater reduction in μ-opioid receptor activation is required before withdrawal occurs. In this simplified modeling exercise, *w* represents withdrawal susceptibility and therefore incorporates various individual components related to for example, chronic opioid exposure, genetics and neuroadaptation (see Discussion) that are not explicitly modeled. R_0_ and *w* represent distinct dimensions: the acute opioid state and susceptibility to withdrawal induced by an opioid antagonist, respectively. In opioid-naive individuals, the probability of withdrawal is zero by definition.

### Utility function

Following Boom et al. (2013) we used the benefit minus harm concept. Where Boom et al. considered analgesia versus respiratory depression (RD), we defined utility here as the probability of respiratory reversal minus the weighted probability of precipitated withdrawal:

$$U=P\left(\text{RD }<\;0.5\right)-k\cdot P\left(\text{withdrawal}\right)$$ (7)

where k denotes the relative weight assigned to withdrawal probability. A value of k = 1 indicates equal weight given to the probabilities of reversal and withdrawal and is used as reference value; it does not imply that respiratory rescue and precipitated withdrawal are equivalent outcomes. Values of k < 1 prioritize reversal relative to withdrawal and values of k > 1 places greater numerical emphasis on avoiding withdrawal. It is generally accepted that k values <1 are most appropriate in clinical practice (*cf.* Gold et al., 2025).

The probability of respiratory reversal, P(RD < 0.5), is determined by the effective fentanyl concentration (C_EF_) and the distribution of C_50_. Because C_EF_ is itself a function of C_E_, C_NALE_ and K_i_, the respiratory reversal depends indirectly on these pharmacokinetic parameters. Withdrawal, P(withdrawal), is evaluated from the μ-opioid receptor activation model and depends on R_0_, R and *w*.

Utility was computed by Monte-Carlo simulations (40,000 virtual subjects per scenario). Individual C_50_ values were sampled from the population distribution, whereas all other parameters were fixed at their typical values because inter-individual variability could not be estimated from available data. The utility was evaluated as a function of steady-state naloxone concentration (0.1–300 ng/mL). Sensitivity analyses examined the influence of withdrawal threshold (*w*), withdrawal probability weight (k), opioid tolerance (C_50_), and overdose severity.

All simulations were implemented in Python 3 using NumPy and SciPy; figures were produced with Matplotlib. A Large Language Model (Claude Opus 4.8, Anthropic, San Francisco, CA) assisted in code development. The study itself is not an artificial-intelligence model; model structure, parameterization, validation, and interpretation were performed by the authors, who take full responsibility for the work.

## Results

### Respiration model

**FIGURE 2:**
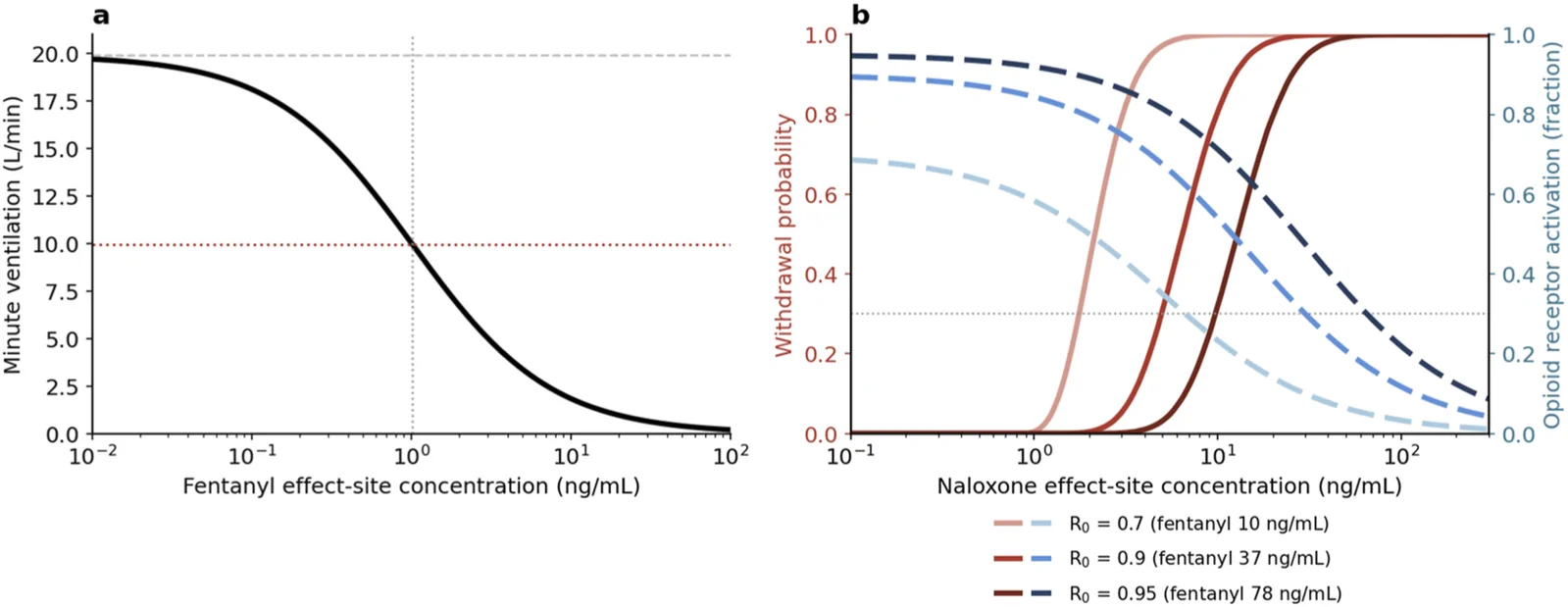
**(a)** Model output of the fentanyl effect-site (steady-state) concentration versus minute ventilation. **(b)** Naloxone effect-site concentration versus receptor activation (right y-axis; blue curves) and versus withdrawal probability (left y-axis; red curves). Three curves are shown with different baseline (pre-naloxone) μ-opioid receptor activation: 0.7, 0.9 and 0.95.

The model reproduced the expected sigmoidal relationship between fentanyl effect-site concentration and ventilation (Figure 2a). Increasing fentanyl concentrations progressively reduced minute ventilation, with the OUD-specific C_50_ (4.08 ng/mL) producing the concentration-response curve used in all subsequent analyses.

#### Withdrawal model

Withdrawal probability increased with increasing naloxone concentrations according to a population-level sigmoid, reflecting progressive reductions in normalized µ-opioid receptor activation (Figure 2b). Higher baseline μ-opioid receptor activation (R_0_) shifted the withdrawal probability curves to the right, indicating that higher naloxone concentrations are required for the same degree of withdrawal (Figure 2b).

### Utility model

**FIGURE 3:**
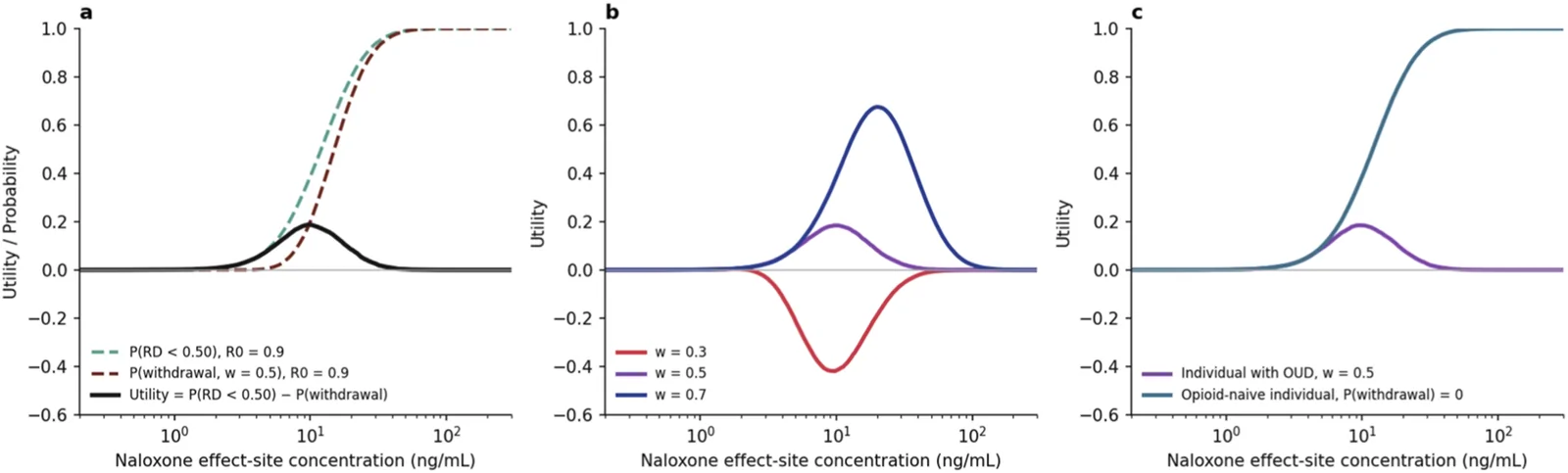
**(a)** Probabilities for reversal of respiratory depression (green broken line) and withdrawal (red broken line) versus naloxone concentration. The black continuous line is the Utility = P(RD < 0.5) – P(withdrawal). Here *w* = 0.5 and R_0_ = 0.9. **(b)** Utility function for *w* = 0.3, 0.5 and 0.7, and R_0_ = 0.9. **(c)** Comparison of the utility in an individual with an OUD and an opioid-naïve individual.

Figure 3a shows the probability of respiratory reversal, the probability of withdrawal, and the resulting utility for R_0_ = 0.9, *w =* 0.5 and k = 1 (Equations 1–7). As naloxone concentration increased, both the probability of respiratory reversal and the probability of withdrawal increased. Their difference yielded a positive numerical utility over a limited effect-site concentration range of approximately 10–30 ng/mL, indicating that the respiratory reversal was more likely than withdrawal within this interval. Maximum utility (+0.2) occurred at an effect-site naloxone concentration of approximately 10 ng/mL.

### Sensitivity analyses

*Withdrawal threshold (w).* The withdrawal threshold strongly influenced the utility function (Figure 3b and Equation 7). At *w* = 0.3, the utility was negative across the concentration range 10–30 ng/mL and zero at values <10 and >30 ng/mL. In contrast, *w* = 0.5 and *w* = 0.7 produced positive peak utilities of +0.2 and +0.7, respectively, reflecting respiratory reversal occurring before withdrawal (Figure 3a). Figure 3c illustrates the comparison between opioid-tolerant and opioid-naïve individuals, the latter exhibiting only beneficial effects because withdrawal cannot occur by definition.

**FIGURE 4:**
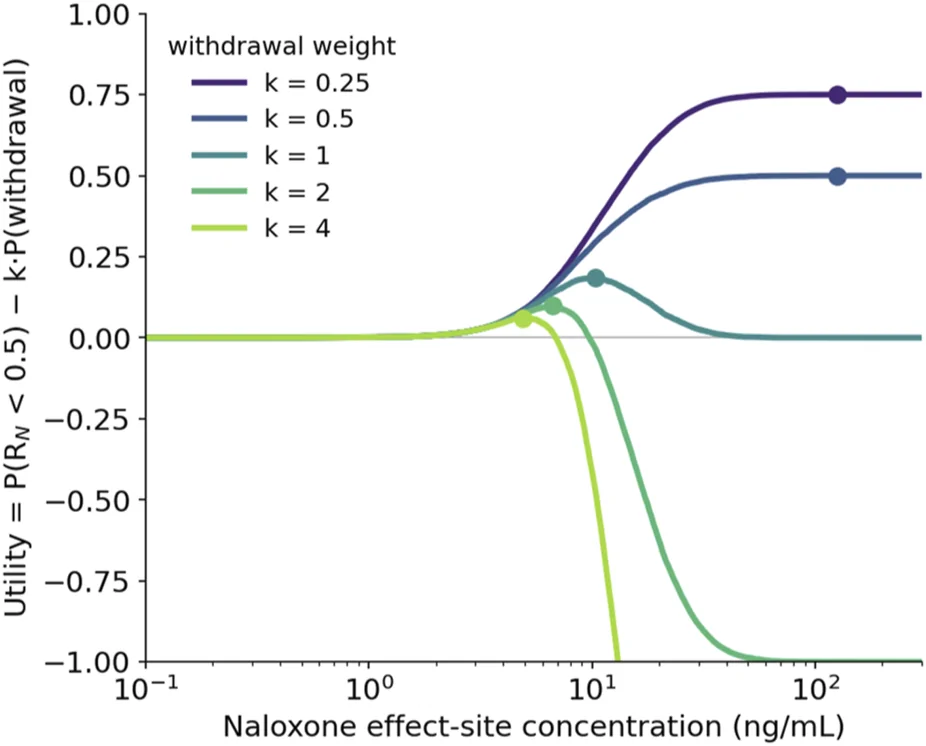
Influence of weight factor k on the utility function. The dots on the curves represent the peak utility values.

*Weight factor k.* Changing the withdrawal probability weight altered the balance between benefit and harm without affecting the probability of withdrawal itself (Figure 4a). Increasing k placed greater emphasis on avoiding withdrawal, whereas k < 1 prioritized respiratory reversal.

*Opioid tolerance (C*_*50*_ *).* For a fixed baseline μ-opioid receptor activation (R_0_), the utility function was independent of C_50_. Greater opioid tolerance required proportionally higher fentanyl concentrations to achieve the same effect, affecting respiratory reversal and withdrawal equally.

*Overdose severity.* The relationship between utility and withdrawal threshold depended on baseline μ-opioid receptor activation, R_0_. At R_0_ = 0.9, the utility function crossed zero at w* = 0.44, *i.e.,* values >0.44 will produce positive utility peaks. More generally the crossover point followed:

$$w*=1-0.5/R_{0}$$ (8)

yielding crossover values of 0.38 at R_0_ = 0.8 and 0.47 at R_0_ = 0.95. Because more severe overdoses produce higher baseline receptor activation (R_0_; Figure 2b), increased opioid doses require progressively greater reductions in μ-opioid receptor activation before respiratory reversal outweighs withdrawal. We express the crossover threshold by w*, representing the withdrawal threshold at which utility changes sign from negative to positive (or *vv*). Since we may assume that the withdrawal threshold (*w*) varies between individuals, those individuals with values below the crossover value of w* (*i.e.,* w < w*) would be expected to experience precipitated withdrawal before respiratory reversal predominates.

## Discussion

Precipitated withdrawal following naloxone administration can be highly distressing and may affect acceptance of treatment or willingness to remain under medical supervision after reversal (Gaddis and Watson, 1992; Buajordet et al., 2004; Neale and Strang, 2015; Monroe and Radke, 2023). However, in life-threatening opioid overdose, rapid restoration of ventilation and survival are more important than withdrawal, consistent with the RESPIRE Expert Forum (Gold et al., 2025). Few studies have related overdose severity and naloxone exposure to withdrawal risk (Yugar et al., 2023; van Lemmen et al., 2025b; van Lemmen et al., 2026), and none, to our knowledge, have explicitly modeled respiratory reversal and withdrawal within a single framework.

To address this gap, we developed a mechanistic PK/PD simulation model based on a utility function, in which model-defined respiratory reversal and precipitated withdrawal are represented simultaneously. Utility analysis has previously been used in pharmacology to quantify desired and adverse drug effects, including analgesia versus respiratory depression and analgesia versus neurocognitive adverse effects (Boom et al., 2013; Moss et al., 2023). Here, we extend this approach by combining an established ventilatory PK/PD model with a novel mechanistic model of withdrawal. Unlike previous utility analyses that directly compared sigmoid E_MAX_ concentration-effect relationships, our model derives withdrawal mechanistically from normalized μ-opioid receptor activation and competitive receptor antagonism. The resulting utility should therefore be understood as an experimental modeling concept rather than a measure of clinical benefit. It describes how the probabilities of two consequences of μ-opioid receptor antagonism change with naloxone exposure (it does not equate their clinical consequences).

The withdrawal model (Equations 1–8) is intentionally simple and contains a single free parameter, the withdrawal threshold *w*, which determines the reduction in normalized μ-opioid receptor activation required to precipitate withdrawal. For example, assuming a baseline activation of R_0_ = 0.9, *w* = 0.3 predicts withdrawal once receptor activation falls below 63% (Equation 6), whereas *w* = 0.5 requires activation to fall below 45%. Importantly, R_0_ and *w* represent different concepts: R_0_ describes the acute opioid effect immediately before naloxone administration, whereas *w* describes susceptibility to withdrawal. Physical dependence is therefore not inferred from acute receptor activation. Rather, *w* is a phenomenological parameter that depends on numerous individual patient-related factors, such as duration of chronic opioid exposure, neuroadaptative changes in the brain related to opioid use, opioid type, recent abstinence, polysubstance use and genetic factors. The distinction between R_0_ and *w* allows, conceptually, that severe acute opioid effect (high R_0_) occurs without withdrawal susceptibility (high value of *w*) and substantial withdrawal susceptibility (low value of *w*) occurs at a lower acute receptor activation (low R_0_). A future model could introduce additional variables, such as a factor that describes the effect of neuroadaptation, but at present available clinical data do not permit reliable parameterization of such models.

**FIGURE 5:**
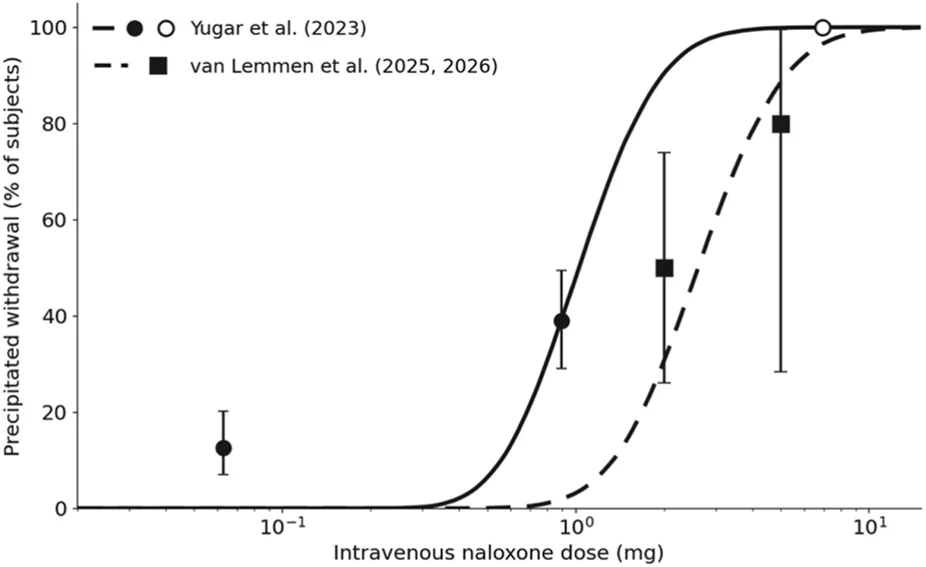
Calibration of the withdrawal set-point against reported naloxone dose–withdrawal data. Continuous and broken lines are the joint maximum-likelihood fit. Data are mean values from the literature ±95% CI.

As given by Equation 8, the utility changes sign at the setpoint w* = 0.44 when R_0_ = 0.9 and k = 1. Because *w* is not directly measurable, we calibrated it by using data from published emergency department cohorts and two prospective naloxone studies (Yugar et al., 2023; van Lemmen et al., 2025b; van Lemmen et al., 2026). This resulted in an estimated withdrawal threshold of 0.30 (95% CI 0.16–0.40) and baseline receptor occupancies of approximately 90% and 96%, respectively. Figure 5 shows the fitted curves for the emergency-department data and daily opioid-user data. If further studies show that the population value of *w* = 0.3 is indeed correct, our experimental model at k = 1 suggests that withdrawal may be more probable than model-defined respiratory reversal over part of the naloxone concentration range, while at values of k < 1, the probability of reversal will exceed that of withdrawal (Figure 4).

In the real-world opioid overdose setting, return of adequate ventilation, prevention of hypoxic injury, and survival take precedence over avoidance of withdrawal (Gold et al., 2025; Thrasher et al., 2026). Treatment is setting-dependent. In community overdoses, concern about withdrawal should never delay administration of available naloxone and activation of emergency medical services and initiation of cardiorespiratory support. In monitored Emergency Medical Services or hospital settings, titration to restoration of adequate ventilation may be feasible while limiting abrupt withdrawal, with ventilatory and/or airway support and treatment of withdrawal when necessary. In case of a cardiac arrest, cardiorespiratory resuscitation take priority and should not be delayed while awaiting a response to naloxone (Thrasher et al., 2026).

Several limitations of our modeling exercise should be acknowledged. First, the withdrawal model is intentionally simplified. Precipitated withdrawal is defined by a threshold parameter rather than by explicit modeling of the complex biology of physical dependence (see above). We deliberately developed this simplified model as available data do not permit reliable parameterization of the underlying withdrawal processes. In the present model, *w* should therefore be interpreted as a measure of withdrawal susceptibility rather than as a direct measure of any single biological mechanism. Future studies should aim at the development of more complex models that include the various components of withdrawal.

Second, the withdrawal threshold was calibrated using heterogeneous, relatively small clinical and experimental datasets that were not originally designed for quantitative PK/PD modelling. Consequently, the estimated value should be regarded as preliminary and hypothesis-generating rather than definitive. Future fentanyl and naloxone concentration-controlled studies with standardized withdrawal assessments will allow more precise estimation of this parameter. We are currently conducting such a study (NCT05338632).

Third, benefit was defined solely as reversal of respiratory depression (RD < 50%). Other possibly equally clinically relevant endpoints include respiratory rate, tidal volume, restoration of hypercapnia and/or hypoxia, prevention of cardiac arrest and/or survival with/without neurological injury. We plan to develop utility functions with cardiac arrest data, based on the Mann et al. (2022) modeling study.

Finally, the present simulations were based on fentanyl overdose and intravenous naloxone and do not represent the full complexity of a current-day overdose. Real-world fentanyl overdoses often involve multiple illicit substances (*e.g.*, benzodiazepines, xylazine) with specific cardiorespiratory and neurological effects that may not be reversed by naloxone. When treating a polysubstance overdose with naloxone (or any other opioid receptor antagonist), persistent respiratory depression or impaired consciousness do not necessarily indicate inadequate naloxone effect. Furthermore, further escalating naloxone doses may increase the probability of withdrawal without restoring breathing.

Our *in silico* modeling has practical advantages. Simulations allow the systematic exploration of dose, concentration, overdose severity, and patient-specific parameters that would be difficult or ethically unacceptable to study experimentally, particularly when severe respiratory depression and precipitated withdrawal are involved. Modeling can therefore reduce the experimental risks and help develop hypotheses for future clinical studies. However, simulations cannot replace clinical studies. Simulation outcome depends on the model structure and assumptions and on the quality of the underlying data. The current model should therefore be viewed as hypothesis-generating.

In conclusion, we developed a mechanistic withdrawal model, obtained a preliminary estimate of the withdrawal threshold *w*, and integrated the probability of precipitated withdrawal with the probability of reversal of respiratory depression. The model provides a quantitative framework for studying these two consequences of naloxone exposure and may help characterize naloxone dosing strategies, compare opioid antagonists, and evaluate approaches that improve ventilation while limiting abrupt withdrawal.

## Data Availability

The original contributions presented in the study are included in the article/supplementary material, further inquiries can be directed to the corresponding author.
